# Editorial: SIRT Family in Endocrinology

**DOI:** 10.3389/fendo.2019.00347

**Published:** 2019-05-31

**Authors:** Tian Li, Fan Peng, Russel J. Reiter, Yang Yang

**Affiliations:** ^1^Key Laboratory of Resource Biology and Biotechnology in Western China, Ministry of Education, Faculty of Life Sciences, Northwest University, Xi'an, China; ^2^School of Basic Medicine, The Fourth Military Medical University, Xi'an, China; ^3^Department of Cellular and Structural Biology, UT Health Science Center, San Antonio, TX, United States

**Keywords:** silent information regulator, endocrinology, diabetes, cardiovascular medicine, metabolism

Silent information regulator (SIRT), also known as NAD-dependent deacetylase sirtuin, is a member of the class III group of histone deacetylases, collectively called sirtuins. The mammalian sirtuin family consists of 7 members, designated SIRT1 through SIRT7, which are characterized by a conserved 275-amino-acid catalytic core and unique additional N-terminal and C-terminal sequences of variable length. Previous studies have demonstrated that SIRT can deacetylate a range of transcription factors, including forkhead box O (FOXO) transcription factors, p53, nuclear factor-κB (NF-κB), liver X receptor (LXR), peroxisome proliferator-activated receptor γcoactivator-1α(PGC-1α), cAMP-responsive element-binding protein–regulated transcription co-activator 2, and period homolog 2 (1).

It has been reported that SIRT performs a wide variety of functions in human systems, including obesity-associated metabolic diseases, endocrine disease, cancer, and aging. Activated SIRT1 improves the insulin sensitivity of liver, skeletal muscle, and adipose tissues and maintains the homeostasis of function and cell mass among pancreatic β-cells, suggesting that SIRT1 might be a new therapeutic target for the prevention of insulin resistance related disease, e.g., metabolic syndrome and type 2 diabetes mellitus (2). In addition, adipose triglyceride lipase (ATGL)-mediated SIRT1 activation promotes autophagy/lipophagy as a primary mean to control hepatic lipid droplet (LD) catabolism and fatty acid (FA) oxidation (3). In mammals, SIRT1 can deacetylate and thereby deactivate the p53 protein. SIRT1 also stimulates autophagy by preventing acetylation of proteins (via deacetylation) required for autophagy as demonstrated in cultured cells, embryonic, and neonatal tissues, which provides a link between sirtuin expression and the cellular response to limited nutrients due to caloric restriction (4). Furthermore, SIRT1 is shown to deacetylate and affect the activity of both members of the PGC1-alpha/ERR-alpha complex, which are essential to metabolic regulatory transcription factors (5).

The research topic covers the themes of diabetes, thyroid diseases, cardiovascular metabolism, cancer endocrinology, bone metabolism. Liu et al. found that estrogen 17β-estradiol (E2) induces CD34 and downregulates SIRT1 in primary mouse airway smooth muscle cells (ASMCs). Then they showed that loss of CD34 inhibits E2-induced reduction of SIRT1 and its enzymatic activity (measured by p53 deacetylation), demonstrating that E2 downregulates SIRT1 through CD34. Since acetylated p53 is well-established to induce apoptotic cell death, the authors further investigated how the E2/CD38/SIRT1/p53 axis affects hypoxia-induced apoptosis. Hypoxia is shown to stimulate CD38/SIRT1/p53 axis (although there is a discrepancy between mRNA levels and protein levels), and thereby E2 promotes hypoxia-induced apoptosis (17β-estradiol promotes apoptosis in airway smooth muscle cells through CD38/SIRT1/p53 pathway). Zhou et al. found the importance of L-serine in decreasing food intake and ameliorating oxidative stress and inflammation response in the hypothalamus of aging mice. The authors fed the aging-mouse models with different concentrations of L-serine, and found its crucial ability to prevent food intake and age-related body weight gain through regulating the leptin pathway and the orexigenic neuropeptides neuropeptide Y (NPY) and agouti-related neuropeptide (AGRP). In addition, they revealed the anti-oxidative and anti-inflammatory role of L-serine, supported by the decreased levels of reactive oxygen species and pro-inflammatory cytokines (IL-1β and IL-6), which is significantly regulated by the SIRT1 and NFκB pathways (Long-term L-serine administration reduces food intake and improves oxidative stress and SIRT1/NFκB pathway in the hypothalamus in aging mice). Moreover, Xu et al. summarized how SIRT1 in the brain controls systemic metabolic homeostasis and then discussed the role of SIRT1 in regulating mitochondrial functions and promoting neuroprotection in the context of cerebral ischemia and neurodegenerative disorders (Brain SIRT1 mediates metabolic homeostasis and neuroprotection). In addition, Yamamoto and Takahashi demonstrated the role of SIRT1 in the hypothalamic pituitary axis and its pathophysiological significance, showing that SIRT1 is involved in the regulatory mechanism of hypothalamus-pituitary axis with respect to the homeostasis maintenance (The essential role of SIRT1 in hypothalamic-pituitary axis).

In addition, Elibol and Kilic provided an overview regarding the association of the increasing level of SIRT1 protein for regulating some disease related conditions such as obesity, cardiovascular diseases, and neurodegeneration as well as some of the functional partners of SIRT1 (High levels of SIRT1 expression as a protective mechanism against disease-related conditions). Zhong et al. introduced the protective roles of SIRT1 in diabetic kidney disease (DKD). The mechanisms of actions are described, highlighting confirmatory results from mice models with DKD. Several SIRT1 activators are discussed as possible therapeutics to attenuate DKD, including resveratrol, puerarin, a novel specific SIRT1 agonist (BF175), and a bromodomain inhibitor (Sirt1 is a potential drug target for treatment of diabetic kidney disease). The present review article by Sergi et al. provides an overview of a number of molecular mechanisms involved in the etiology of osteosarcoma. They tried to clarify the interaction among insulin/IGF-1R, FOXO, and SIRT1 pathways, further explicating their roles in bone-related diseases. Specifically, the authors focus on the FOXO family of transcription factors (Insulin/IGF-1R, SIRT1, and FOXOs pathways–an intriguing interaction platform for bone and osteosarcoma). In addition, Fujita and Yamashita discussed the roles of the sirtuin family of proteins especially SIRT1 in pathological and physiological conditions of the central nervous system and focused their attention on the potential benefits of activators of SIRT1 in neurodegenerative diseases (Sirtuins in neuroendocrine regulation and neurological diseases). Zhang et al. discussed the predicted and validated roles of SIRT1 in ischemic stroke and potential stroke therapeutics, with a significant focus on Chinese medicines (The role of SIRT1 in ischemic stroke: pathogenesis and therapeutic strategies). Moreover, Liarte et al. aimed to conclude the available presentation on the interplay between SIRT1 and ER/GPER for breast cancer onset and progression. They discussed the ability of SIRT1 to interact with TGFß signaling, a concurrent signaling significantly involved in breast cancer progression, which will benefit for the development of innovative strategies in the assessment of orphan breast cancer subtypes, such as triple negative breast cancer (TNBC) (SIRT1 and estrogen signaling cooperation for breast cancer onset and progression). Frazzi described the role of SIRT1 in cancer with special emphasis on secretory organs' cancer. They sought to summarize the association between SIRT1 overexpression and poor overall survival in patients with liver and lung cancers, and no association with breast cancer, colorectal cancer or gastric carcinoma. The results show that unfavorable overall survival is associated with SIRT1 expression for solid malignancies (SIRT1 in secretory organs' cancer). Artsi et al. reported the role of SIRT1 in marrow adipose tissue (MAT) phenotype, demonstrating its stimulatory effect on a thermogenic gene program in female MAT, showing that SIRT1 activation and blocking sclerostin stimulate a thermogenic gene program in human bone marrow mesenchymal stem cell (BM-MSC) (Sirt1 promotes a thermogenic gene program in bone marrow adipocytes: from mice to (wo)men). Furthermore, Sun et al. confirmed that SIRT1-mediated molecular events and biological processes could be an underlying mechanism for metastasis and SIRT1 is a therapeutic target for inhibiting metastasis (Survival and clinicopathological significance of SIRT1 expression in cancers: A meta-analysis).

In addition to SIRT1, other molecules in the SIRT family also play an important role in endocrine secretion. Zhou et al. presented the recent advances of SIRT1-7 in various insulin resistance-associated physiological processes. In particular, they highlighted the roles of sirtuins in insulin resistance including inflammation, mitochondrial dysfunction, the insulin signaling pathway, glucose and lipid metabolism (Sirtuins and insulin resistance). Song et al. evaluated the roles of sirtuins in diabetes progression and described their involvement in metabolic pathways of skeletal muscle, adipose tissue, and liver. They suggested that SIRT1, SIRT2, SIRT3, and SIRT6 are positive regulators of insulin resistance in most cases while SIRT4 and SIRT7 negatively regulate insulin in secretion and fatty acid oxidation. The identifications of potential pharmacological targets of sirtuins may enhance the understanding on the regulation of glucose metabolism and homeostasis in diabetes (Distinctive roles of Sirtuins on diabetes, protective or detrimental). In addition, Min et al. summarized the current literature on the function of SIRT4, one of the lesser known and studied of the sirtuin family. The review introduces the metabolic functions of SIRT4 in various tissues and its role as a tumor suppressor (The roles of mitochondrial SIRT4 in cellular metabolism). Wu et al. managed to concisely present the current knowledge on the functions and mechanisms of SIRT7 in cellular regulation and disease (Advances in cellular characterization of the sirtuin isoform, SIRT7).

The research topic highlights some of the recent findings about diabetes, thyroid diseases, cardiovascular metabolism, cancer endocrinology, bone metabolism. I would like to thank all the authors and reviewers for their contribution and discussion to put together this wonderful topic that may inspire further interest in this exciting new field.

## Author Contributions

All authors listed have made a substantial, direct and intellectual contribution to the work, and approved it for publication.

### Conflict of Interest Statement

The authors declare that the research was conducted in the absence of any commercial or financial relationships that could be construed as a potential conflict of interest.
